# The multiplicity of divergence mechanisms in a single evolving population

**DOI:** 10.1186/gb-2012-13-6-r41

**Published:** 2012-06-08

**Authors:** Ram P Maharjan, Thomas Ferenci, Peter R Reeves, Yang Li, Bin Liu, Lei Wang

**Affiliations:** 1 School of Molecular and Microbial Biosciences, University of Sydney, NSW 2006, Australia; 2 TEDA School of Biological Sciences and Biotechnology, Nankai University, 23 Hongda Street, Tianjin 300457, PR China; 3 Key Laboratory of Molecular Microbiology and Technology, Ministry of Education, 23 Hongda Street, Tianjin 300457, PR China

## Abstract

**Background:**

Evolutionary divergence is common within bacterial species and populations, even during a single bacterial infection. We use large-scale genomic and phenotypic analysis to identify the extent of diversification in controlled experimental populations and apply these data to differentiate between several potential mechanisms of evolutionary divergence.

**Results:**

We defined testable differences between five proposed mechanisms and used experimental evolution studies to follow eight glucose-limited *Escherichia coli *chemostat populations at two growth rates. Simple phenotypic tests identified 11 phenotype combinations evolving under glucose limitation. Each evolved population exhibited 3 to 5 different combinations of the 11 phenotypic clusters. Genome sequencing of a representative of each phenotypic cluster from each population identified 193 mutations in 48 isolates. Only two of the 48 strains had evolved identically. Convergent paths to the same phenotype occurred, but two pleiotropic mutations were unique to slow-growing bacteria, permitting them greater phenotypic variance. Indeed, greater diversity arose in slower-growing, more stressed cultures. Mutation accumulation, hypermutator presence and fitness mechanisms varied between and within populations, with the evolved fitness considerably more uniform with fast growth cultures. Negative frequency-dependent fitness was shown by a subset of isolates.

**Conclusions:**

Evolutionary diversity is unlikely to be explained by any one of the available mechanisms. For a large population as used in this study, our results suggest that multiple mechanisms contribute to the mix of phenotypes and evolved fitness types in a diversifying population. Another major conclusion is that the capacity of a population to diversify is a function of growth rate.

## Background

Heritable variation is common in evolution and certainly within bacterial species and populations [1,2]. Bacterial divergence is rapid and even evident within the course of a single bacterial infection [3]. One of the most intriguing questions in evolutionary biology is how such variation emerges and persists. It used to be believed that a population evolving in a homogeneous environment should exhibit low sympatric diversity as a result of periodic selection of a fitter isolate and the competitive niche exclusion of weaker isolates [4]. Therefore, environmental heterogeneity was considered to be the main source of diversity [5]. However, several experimental evolution studies suggest that diverse types coexist even in an initially homogeneous culture supported by a single resource [6-8].

Several distinct mechanisms or explanations have been proposed for the maintenance of diversity in evolving populations, including frequency-dependent selection [9,10], cross-feeding and cheating between population members [11,12], metabolic and other trade-offs [13,14], mutation selection balance [15,16] and regulatory degeneracy leading to convergent evolution [7,17]. The multiplicity of mechanisms begs the question: what happens in real diversifying populations? Do individual populations predominantly diversify by one of the above mechanisms or is evolved diversity a result of a mixture of mutation-selection balance, frequency-dependent selection, trade-offs with or without mutation rate differences, niche creation or convergent evolution through degenerate pathways? The predictability and a systems-level description of evolution [18] require an answer to this question.

To approach the above questions, the signatures of individual divergence mechanisms need to be searched for in the evolved isolates. It is also necessary to obtain an accurate characterization of the extent of diversity in populations at both phenotypic and genotypic levels. Bacterial cultures, with their large populations, rapid growth and regulated mutation rates should be ideal subjects for the experimental analysis of evolution and diversification [5,18,19]. Genomic diversity can be readily identified through genome sequencing, as has been done in several experimental populations [7,17,20,21]. However, owing to the difficulty of identifying phenotypic diversity, most previous experimental studies focused on a few adaptive isolates or mutations and no experimental study has systematically characterized the extent of diversity at both phenotypic and genotypic levels in multiple parallel populations. Also on ecological grounds, studies of diversity in experimental populations have tended to use culture conditions with multiple or alternative niches [22-27]. Even the intensively studied Lenski populations evolved in a selection environment that fluctuated temporally in terms of nutrient availability through multiple cycles and coexisting types have evolved to these fluctuations [28]. Recent genomic analyses of these long-term populations have revealed multiple mutations [20]. The beneficial effects of these mutations are beginning to be revealed [29,30], but many of the mutations have unidentified benefits or fitness effects. A systematic description of diversity in these populations has not been reported, although metagenomic studies indicate diversity does exist in the Lenski populations. The detection of minor types was limited by the resolution of the metagenomic technique [20]. In contrast, we showed that extensive diversification can be detected when extensive phenotypic screening is applied to populations, and described in detail the complex population structure of a single chemostat population [6].

We report a study that relates the experimental results on eight evolving populations to the above divergence mechanisms. The phenotypic testing permitted the identification of multiple phenotypic groupings, and fitness assays were applied to test for trade-offs and frequency dependence. A detailed analysis of genomic changes revealed whether the number of mutations in each isolate is significantly different. These data are used to discuss whether mutation-selection balance [15,16] or trade-offs with differences in mutational robustness [14] or regulatory degeneracy leading to convergent evolution [7,17] are involved in the mechanism of diversification. We therefore compare the number of mutations per isolate in both sympatric and allopatric populations. In addition, we analyzed all 320 isolates compared here for mutator activity, because hypermutation can be selected in evolving populations [31,32]. We indeed find that two of eight populations contain a subset of isolates with elevated mutation rates. Our strain comparisons also address the proposal that convergent alternative mutational pathways lead to similar levels of fitness in the same culture [17,33]. We also followed frequency dependence in fitness assays and looked for mutations known to cause altered trade-offs, such as *hfq *and *rpoS*, that reset the trade-off between stress protection and nutritional competence (SPaNC) [13,34]. The availability of 48 genome sequences further allows us to detect patterns of mutations as well as simultaneous analysis of phenotype-genotype relationships in a large number of isolates. Altogether, the numbers of mutations responsible for multiple phenotypes provide for the first time a comprehensive analysis and clues to the kind of mechanisms and properties of isolates relevant to the various evolutionary models.

## Results

### Evolution of phenotypic diversity in parallel populations

In order to test how diversity emerges and is maintained in bacterial populations, we performed replicate evolution experiments using glucose-limited chemostat cultures of *Escherichia coli *at two separate growth or dilution rates (D); four at slow with D = 0.1 h^-1 ^(generation time of approximately of 6.9 hours) and four at fast with D = 0.6 h^-1 ^(generation time of 1.15 hours). These two conditions result in a 30-fold difference in basal mutation frequencies per generation [35] and this difference is important for determining the mechanisms of diversification; the two series of experiments test whether the extent of diversity changes under these otherwise near-identical selection conditions. All chemostats were initiated with a common ancestral strain, BW2952 [36], and the population size was maintained at approximately 1.6 × 10^10 ^cells by a constant concentration of glucose in the feed medium throughout the evolution experiment. The cell density did not vary appreciably during the course of the experiments.

**Figure 1:**
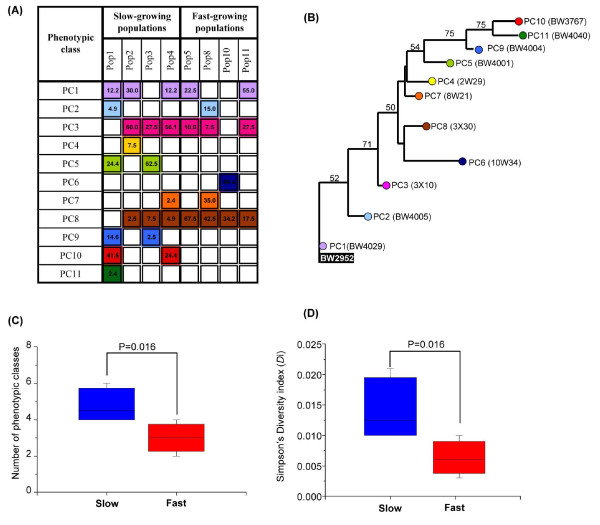
**Phenotypic diversity in *E. coli *populations evolving in glucose-limited chemostats**. **(a) **The sympatric divergence of at least 39 isolates randomly picked from each population grown at slow-dilution rate (0.1 h^-1^; populations (Pop) 1 to 4) and fast-dilution rate (0.6 h^-1^; populations 5, 8, 10 and 11) were based on three characteristics: iodine staining of colonies, sensitivity to methyl α-glucoside (α-MG) and *malG-lacZ *expression level (fold- change in Miller units relative to ancestral strain). The individual properties of each test are listed in Table S1 in Additional file 1 and the criteria used for defining the phenotypic classes (PCs) are shown in Table S2 in Additional file 1. Each of 11 identified PCs 1 to 11 are marked with different colors. Numbers in the colored boxes indicate the frequency (percentage) of the isolate in the population. Boxes without colors indicate that the corresponding PCs are absent from the population. **(b) **Relationships of the 11 PCs were obtained by the neighbor-joining method rooted to the ancestral strain, BW2952 (black box white type), as described in PAST [69]. The dendrograms were based on the three characteristics described in (a). Each terminal branch represents a phenotypic class (PC) and letters in parentheses indicate the individual phenotypic property belonging to the PC (+, wild-type iodine staining; -, no staining; P, partial staining; S, sensitive to α-MG; R, resistance to α-MG) and numbers 1 to 10 in parentheses indicate *malG-lacZ *expression level (fold changes in Miller units relative to ancestral strain). The bootstrap values at the branches are percentages based on 1,000 replicates. **(c) **The number of phenotypic classes in slow-dilution rate or 0.1 h^-1 ^and fast-dilution rate or 0.6 h^-1 ^populations. The phenotypic classes were based on (a), in which each colored box represents one PC. **(d) **The level of phenotypic diversity in 0.1 h^-1 ^and 0.6 h^-1 ^populations presented as the Simpson's diversity index (*Di*) of four replicate evolving populations from each dilution rate. The difference in *Di *was significant (two-tailed *t*-test, *P *= 0.033). The error bars are the standard deviation based on replicate populations.

To determine the phenotypic diversity in evolving populations, 39 to 41 isolates from each of the 8 independent chemostat cultures were analyzed. Population 1 was used previously and the properties of its 41 isolates reported [6]. The samples were obtained from the last day before a particular chemostat malfunctioned or was terminated, between days 23 and 26, except for population 4, which stopped at day 18. Isolates were randomly chosen and characterized using the triple-phenotypic-test approach (see Materials and methods). This method identifies the six phenotype clusters in our best-analyzed population and detects the major population fluctuations that occur in the first two weeks of culture [6]. Co-existence of four or more combinations of phenotypes was evident after two to three weeks in this earlier study [6] and population diversity did not change markedly between days 15 and 37, as shown in Figure S1 in Additional file 1. So samples from the eight populations at days 21 to 27 provide a good snapshot of population heterogeneity. We may underestimate the extent of diversity in this way, but as shown in Figure 1a, significant heterogeneity was detectable in a single sample from all eight cultures. We found two to six combinations of phenotypic classes (PCs) in each population (Figure 1a). Individual characteristics of all analyzed isolates are shown in Table S1 in Additional file 1 and criteria used for classification of each PC are shown in Table S2 in Additional file 1.

**Table 1:** Quantification of mutations in slow- and fast growing cultures

	SNPs		Indels		SNP/indel ratio	
			
	0.1 h^-1^	0.6 h^-1^	0.1 h^-1^	0.6 h^-1^	0.1 h^-1^	0.6 h^-1^
Coding	22	17	5	10	4.40	1.70
Intergenic	5	6	8	10	0.63	0.60
Total	27	23	13	20	2.08	1.15

Note: only mutations from non-mutators are used for comparison.

Altogether, 11 different PCs were distinguishable in the eight populations (Figure 1a). The extent of the diversity and the phenotypic relationships of the 11 PCs is shown in Figure 1b. Most, but not all, of the PCs were found in both slow- and fast-dilution rate cultures, although none were universally present. PC1 (ancestor class), PC3 and PC8 were present in five, six and seven cultures, respectively, making them the most widely distributed classes (Figure 1a). Some PCs were present only with a particular dilution rate; for example, PC 5 and PCs 9 to 11 were absent in fast-dilution rate cultures while all PCs except PC6 were found in slow-dilution populations. As shown in Figure 1c, on average, there were 4.75 PCs in 0.1 h^-1 ^cultures compared to 3.0 in 0.6 h^-1 ^cultures, which suggests that the slow-dilution cultures became more diverse than the fast-dilution cultures, despite dividing for one-sixth of the number of bulk generations but with higher mutation rates. Simpson's diversity indices (*Di*) also indicated that populations from the slow-dilution cultures were more diverse (*Di *= 0.0282) compared to those from fast-dilution cultures (*Di *= 0.0134) (Figure 1d; two-tailed *t*-test *P *= 0.0329). Hence, both the types of PC and the overall diversity in evolution over the same elapsed time are influenced by small differences in evolutionary selection conditions, in this case residual glucose concentration determining growth rate in the chemostat.

In addition, the proportion of individual PCs varied between slow and fast dilution populations. PCs 5 and 10 are the most abundant classes in the slow-dilution populations while PCs 8 and 6 accounted for more than 50% of the total isolates analyzed from the fast-dilution populations. Some low-abundance types were unique to one population (for example, PC11 in population 1 (Figure 1a)). Some of these differences may be due to a focus on a single time point or limited sampling. However, in the one population for which we analyzed the population structure over a greater period (over 37 days) we found slightly changing proportions beyond day 27 due to continuing population turnover, but no major sweeps (Figure S1 in Additional file 1). Overall, there appears to be no sympatric or allopatric uniformity between the populations when subject to a similar, relatively homogeneous, constrained selection condition. In common with studies on another experimental system [27], unparallel diversification is an important feature of bacterial evolution.

### Mutations in evolved genomes

To investigate the genetic basis of phenotypic diversity and which mechanism(s) were responsible for the emergence of diversity, we sequenced the whole genomes of at least one isolate from each PC in all eight populations. Altogether we sequenced 48 isolates, 27 from slow- and 21 from fast-dilution cultures (the strains are listed in Table S3 in Additional file 1). The fully sequenced ancestral genome [36] was used as the template for identifying the mutations in the evolved isolates.

**Figure 2:**
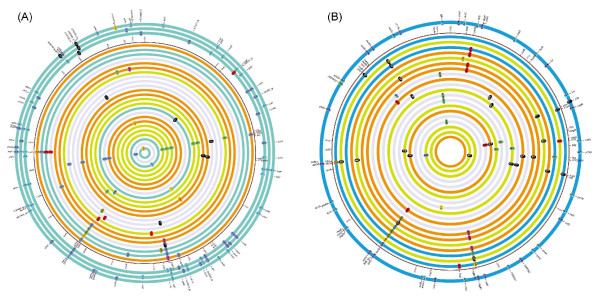
**Mutational changes in the genomes of 48 evolved isolates from slow- and fast-dilution rate populations**. **(a) **Genome changes in the 0.1 h^-1 ^populations. The thin circle shows the map of the ancestor genome with the genes altered in isolates. The populations that the isolates came from are color-coded: population 1 (blue), population 2 (green), population 3 (orange) and population 4 (grey). The outer three circles show the mutations in the three mutator isolates BW4004, BW4040 and W3767 (outermost to innermost) and the other circles represent the following isolates in order towards the center: 3X37, BW4030, BW4002, 4R1, 3X30, 3X27, 2W14, 4R23, 4R17, 4R16, 3X16, 3X10, 2W29, 2W22, BW4005, 4R10, 3X31, 3X17, 2W30, 2W11, 2W2, BW4029, 4R18, BW4001. The mutations are also color-coded, so identical mutations in the same gene share the same color. **(b) **Genome changes in 0.6 h^-1 ^populations. The thin circle shows the map of the ancestor genome with the genes altered in isolates. The populations that the isolates came from are color-coded: population 5 (orange), population 8 (green), population 10 (blue) and population 11 (grey). The outer circle shows the mutations in the mutator isolate 10W34 and the other circles represent the following isolates in order towards the center: 10W40, 8W33, 10W21, 5W3, 8W21, 5W30, 5W19, 11Za3, 5W21, 11Za12, 8W20, 11Za16, 11Za1, 8W18, 5W23, 11Za5, 11Za4, 8W37, 8W17 and 5W25. The mutations are also color-coded, so identical mutations in the same gene share the same color.

We detected a total of 193 mutational events in the 48 evolved isolates (see Figure 2 for a graphic summary and Table S4 in Additional file 1 for a complete list of all identified mutations). There were a total of 155 SNPs, of which 140 were located in protein coding regions and the remaining 21 in intergenic regions. There were also 15 insertions and 23 deletions (indels). The majority of evolved isolates contained one to five mutations except in four isolates (BW3767, BW4004 and BW4040 from population 1 and 10W34 from population 10) that contained 28 to 46 mutations. This greatly elevated rate of genomic mutation was a result of *mutY *gene deletions as all four genomes contained a deletion of approximately 28 kb DNA including *mutY*, consistent with the demonstrated insertion sequence (IS)*5*-mediated rearrangement involved in *mutY *deletions [37]. Lack of *mutY *activity causes C:G > A:T transversions [38] and almost all SNPs in these isolates showed a C:G > A:T signature. To test the overall presence of mutators, we screened all the 321 isolates for the *mutY*-related mutator phenotype by testing their ability to grow on glycolate (the deleted region in all *mutY*-mutator isolates contained the *glc *locus responsible for glycolate utilization [37]) and compared the frequency of spontaneous rifampicin-resistant mutants. We found an additional 20 and 15 isolates with *mutY*-mutator phenotypes from populations 1 and 10, respectively. No isolates with elevated mutation rates were detected in the other six populations.

### Mutational patterns in slow and fast growing cultures

Figure 2 and Additional file 2 indicate that the mutational patterns under the two selection conditions were not identical. In the 44 non-mutator isolates, we found 27 SNPs (22 in coding and 2 in intergenic regions) and 23 SNPs (17 in coding and 6 in intergenic regions) in 0.1 h^-1 ^and 0.6 h^-1 ^populations, respectively (Table 1). All SNPs except one in a fast-growth culture (*metB *in 11Za13; Table S4 in Additional file 1) were non-synonymous (nsSNPs), affecting polypeptide sequence. We used the SIFT algorithm [39] to evaluate the effect of nsSNPs on protein function. All of them were predicted to be non-conservative and therefore expected to exert some effect on protein function. This result is consistent with the predominance of nsSNPs in the recent studies of the *E. coli *populations evolving under different experimental settings [21,40,41], suggesting that most mutations fixed in the early phase of evolution are adaptive.

**Figure 3:**
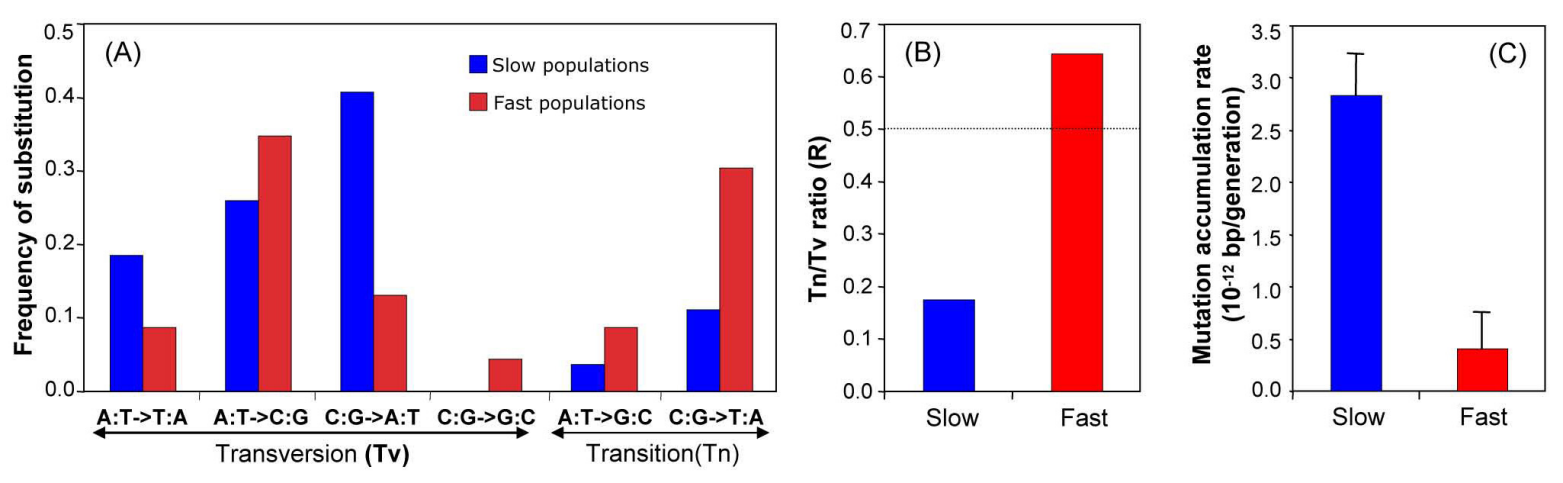
**Mutational bias between slow- and fast-dilution rate populations**. **(a) **Frequency and pattern of different substitutional mutations. **(b) **Ratio between transition and transversion substitutions. **(c) **Mutation accumulation rates were estimated from the average number of observed mutations in slow growing populations, the number of generations, the effective population size and the mean selection coefficient per generation of all sequenced non-mutator isolates (24 from slow and 20 from fast dilution populations), as described in [52]. The error bars are the standard deviation based on replicate populations.

About 85% and 70% of all SNPs in the 0.1 h^-1 ^and 0.6 h^-1 ^populations, respectively, were transversions. When analyzing transitions and transversion events, the ratio was found to be different between the 0.1 h^-1 ^and 0.6 h^-1 ^populations (Figure 3a, b). The ratio was 0.6 in the 0.6 h^-1 ^population, which was slightly higher than the cumulative ratio obtained from sequence comparison of bacterial genomes [42]. By contrast, the ratio of 0.17 in slow-growth populations was nearly three-fold less than the expected ratio of 0.5, indicating that transversion mutations are more common under slow-growing environments; the reason for this skewed profile, together with the higher mutation rates in 0.1 h^-1 ^cultures, needs investigating. However, in line with the Ochman's analysis [42], we observed only one C:G→G:C transversion mutation in all of the 48 genomes, confirming the rarity of this mutation.

We also found strikingly different patterns of indel mutations between the 0.1 h^-1 ^and 0.6 h^-1 ^populations. Among the 31 indels in non-mutator isolates, 12 were single-base while the remaining 19 were large indels. Interestingly, 10 out of 12 single-base indels were in the 0.6 h^-1 ^population. The majority of the large indels (> 1 bp) were associated with IS elements, consistent with the extensive movement of a variety of IS elements demonstrated recently [37]. The SNP-to-indel ratio of 2.08 in 0.1 h-1 populations was nearly two-fold higher than that of 1.15 in 0.6 h-1 cultures, which reflects that indels are more common in 0.6 h-1 populations (Table 1). However, in comparison to other bacteria, the observed SNP-to-indel ratio is significantly lower under both selection conditions, suggesting the importance of indels during constant selection under nutrient limitations. The genome-wide SNP-to-indel ratio in bacterial genome comparisons is estimated at around 19.61 [43].

In addition to differences in mutational patterns, mutation accumulation rates (MARs) were also found to be affected by the growth rate. As shown in Figure 3c, there was approximately a four-fold higher MAR in 0.1 h^-1 ^than in 0.6 h^-1 ^populations (two tail *t*-test *P *= 0.009). The mean MAR in 0.1 h^-1 ^populations was 1.78 × 10^-12 ^mutations per site per generation compared to 0.40 × 10^-12 ^mutations per generation in 0.6 h^-1 ^populations (Figure 3c). These values cannot be compared to real mutation rates, which are approximately 100-fold lower [41,44,45]. Of course, MAR is affected by selection as well as mutation rate as mutation supply is probably not limiting in large populations [46].

### Fitness properties of evolving isolates

**Figure 4:**
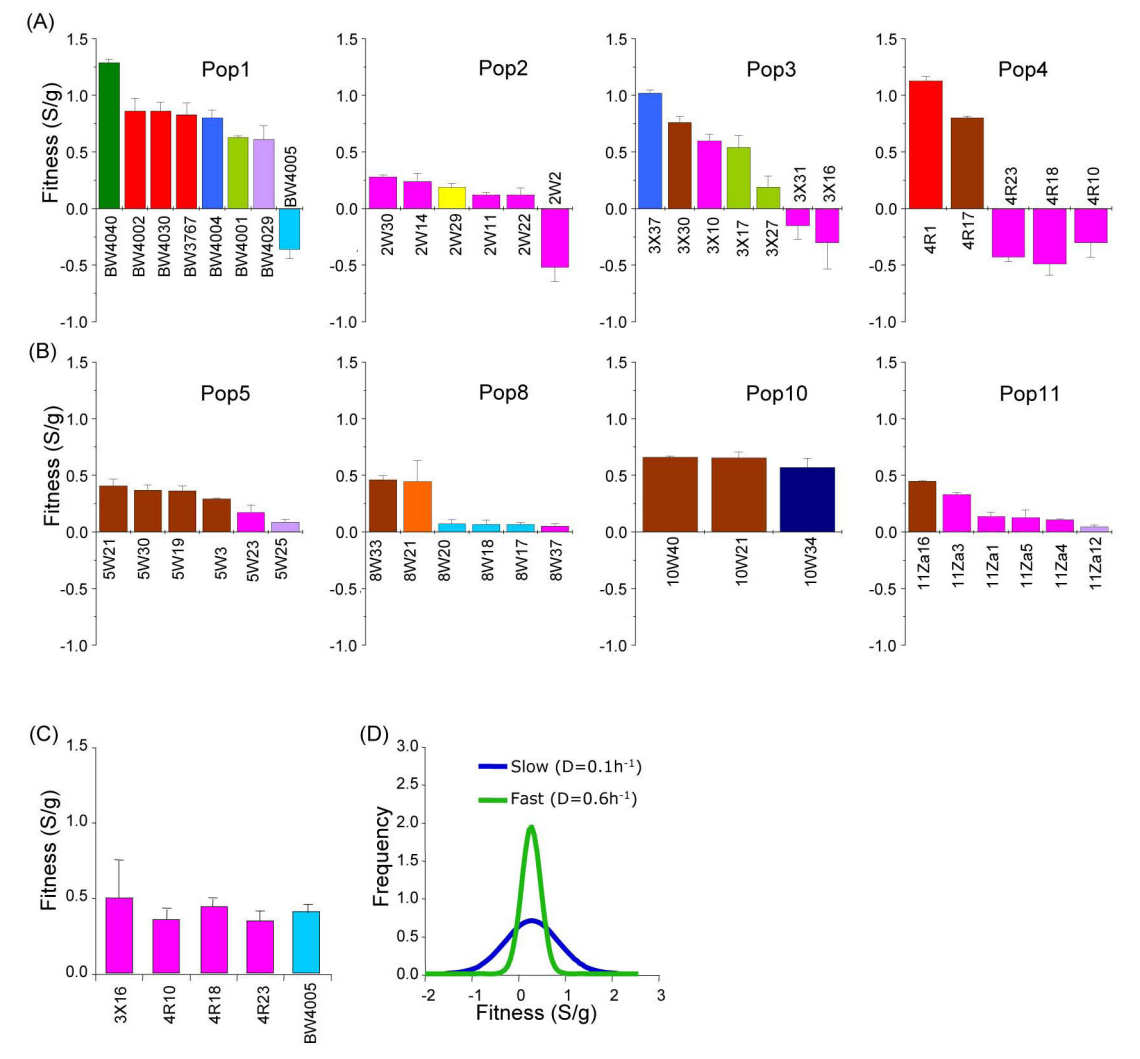
**Distribution of fitness estimates within evolving *E. coli *populations**. All competitions for fitness assay were performed against reference strain BW3454 containing *metC::*Tn*10 *as described in Materials and methods. The reported fitness, S/generation, where S is the selection coefficient (estimated according to [73]). All data presented are mean ± standard deviation from at least three independent experiments. **(a) **Selection coefficients of evolved isolates derived from 0.1 h^-1 ^populations were measured at the same dilution rate after mixing 50/50 with the reference strain. **(b) **Evolved isolates from 0.6 h^-1 ^populations were measured at the same dilution rate after mixing 50/50 with the reference strain. **(c) **Negative frequency-dependent fitness of isolates with negative fitness in Figure 1a were measure after mixing 1/99 with the reference strain. **(d) **Normal distribution of fitness in evolving isolates were estimated from standard deviations and mean fitness (Sg). Standard deviation = 0.57 for slow- and 0.207 for fast-growing populations and Sg = 0.295 for slow- and 0.283 for fast-growing populations.

The nature of the fitness can indicate the means of selection of particular isolates. Thus, identifying the fitness properties of evolved isolates is crucial for understanding the mechanism of adaptation. To estimate the fitness, we measured the selection coefficient of each of the 48 isolates used for genome sequencing by competing head-to-head at a 50:50 initial ratio with the ancestral isolate under the dilution conditions under which they were isolated (see Materials and methods). We showed previously that isolate-to-isolate competition gives a similar rank order of fitness characteristics as isolate-to-ancestor competition [6]. As shown in Figure 4a, b, the majority, but not all, of the isolates from both slow and fast-dilution cultures have a significant competitive advantage against the common ancestor in their respective original selection environments.

Perhaps most interestingly, isolates with significantly reduced fitness compared to the ancestral isolate were present in each of the slow growth populations; these include BW4005 from population 1 and six newly identified isolates, 2W2 from population 2, 3X16 and 3X31 from population 3 and 4R18 and 4R10 from population 4. These isolates may have survived through adaptation to a novel niche created within the original glucose-limited environment and/or negative frequency dependence. In fact, when pair-wise competitions were started at 1% against 99% ancestral isolate, we found that these isolates exhibited a positive fitness against the ancestral clone (Figure 4c). Hence, negative frequency-dependent selection is a feature of several evolving populations, but not all isolates show this property. Interestingly, 2W2, 3X16, 3X31, 4R10, and 4R18 all belonged to PC3 while BW4005 belonged to PC2, indicating that frequency-dependence is not always associated with the same phenotype. There is also diversity in genotypes; as shown in Table S4 in Additional file 1, the frequency-dependent isolates had a variety of mutations. For example, 2W2 contained mutations associated with *csgD*, 3X16, 3X31, 4R10, and 4R18 contained a SNP in *rpoA *(RNA polymerase A) and BW4005 contained an in-frame deletion of 3 bp in *aphC *and *lpxM *and a deletion of *ogrK*-to-*yegS*, suggesting convergence of phenotype through multiple pathways. The molecular mechanism(s) of negative frequency-dependent fitness in these populations has not been identified, but the diverse mutations suggest several alternatives exist.

### Fitness and the growth rate under selection

The data in Figure 4a, b also showed that both the magnitude of fitness gain and the extent of fitness distributions within and across the populations were markedly different between 0.1 h^-1 ^and 0.6 h^-1 ^populations. Amongst the 48 isolates, the PC11 isolate (BW4040), which contained 29 mutations, including in *rpoS, mglD *and *malT*, exhibited the highest fitness relative to the ancestral isolate. The measured fitness (1.29 S/g) was over two-fold higher than that of the most-fit counterparts in the 0.6 h^-1 ^populations, suggesting the fitness peak heights are very different under the two conditions.

Consistent with the phenotypic diversity, the 0.1 h^-1 ^populations exhibited a greater level of fitness diversity than the 0.6 h^-1 ^populations. The standard deviation of measured fitness amongst the 26 0.1 h^-1 ^isolates (0.53 S/g) was more than two-fold higher than the 0.21 S/g value of the 0.6 h^-1 ^populations. The mean fitness gain of 0.36 S/g in the 0.1 h^-1 ^populations was slightly higher that that of 0.3 S/g in the 0.6 h^-1 ^populations. The difference, however, was not significantly different (two-tailed *t*-test *P *= 0.09). Furthermore, the normal or Gaussian distribution of fitness based on the standard deviation and the mean fitness showed that the width of fitness distributions in the 0.1 h^-1 ^populations is broader than in the 0.6 h^-1 ^populations (Figure 4d). These results are notable, particularly in the 0.1 h^-1 ^populations, because the mutation-selection balance proposal (analyzed below in detail) predicts that elimination of less fit isolates through clonal interference depends on the fitness distribution between contending lineages. However, the observed wide distribution of fitness within the 0.1 h^-1 ^populations did not lead to clonal replacement; instead we observed expansion of some less-fit subpopulations in the later samples. For example, we found that isolates from PC9 to PC11 in population 1 have the highest measured fitness but failed to replace members of the PC5 subpopulation (Figure S1 in Additional file 1). In fact, the proportion of PC5 isolates gradually increased from 5% of the total population at day 17 to over 50% at day 37 despite their fitness deficit against the isolates belonging to PC9 to PC11.

These results together with genome and phenotypic data also revealed that, in some cases, fitness convergence was achieved despite having no genotypic or phenotypic similarity. For example, BW4001 and BW4029 in population 1, 2W14 and 2W29 in population 2, 3X10 and 3X17 in population 3, 8W33 and 8W21 in population 8 and 10W21 and 10W34 in population 10 were different at both phenotypic and genotypic levels (Figure 4; Table S4 in Additional file 1) but had similar competitive fitness. These results clearly indicate that it is possible for coexisting subpopulations to achieve the same height of fitness peak through multiple mutational pathways.

### Parallel mutations and genetic basis of the biased phenotypic diversity between 0.1 and 0.6 h^-1 ^populations

To gain further insight into the genetic basis of the phenotypic diversity and to understand which divergence mechanism is responsible for the observed diversity, we identified genes that acquired mutations in parallel populations. Genes repeatedly mutated in more than one population are more likely to contribute fitness. The 193 mutations in isolates were distributed in 165 genes or regions, of which 12 genes were found in more than one population (Table 2). Multiple alleles were found in the majority of repeatedly mutated genes and in almost all cases, the evolved alleles were different between populations. Mutations in *rpoS *and *mglD/O *were the most common, both appearing in six out of eight populations, three each in the 0.1 and 0.6 h^-1 ^populations. Other genes mutated under both selection conditions include *csgD-ig, cobB, inaA_ig, xapA, rpoB *and *rpoA *as well as an approximately 29 kb DNA deletion including *mutY *(Table 2).

**Table 2:** Genes or regions with mutation(s) in parallel populations

		Total number of alleles	Selection conditions	
			
Gene or region	Product		0.1 h^-1^	0.6 h^-1^
*rpoS*	RNA polymerase sigma subunit	12	3 out of 4	3 out of 4
*mglD/O*	DNA-binding transcriptional regulator	9	3 out of 4	3 out of 4
*hfq*	RNA-binding protein Hfq	4	2 out of 4	None
*cgsD_ig*	DNA-binding transcriptional regulator	4	1 out of 4	2 out of 4
cobB*	Deacetylase of acetyl-CoA synthetase, NAD-dependent	2	1 out of 4	1 out of 4
28 to 30 kb with *mutY *deletion*	Not applicable	2	1 out of 4	1 out of 4
rpoB*	DNA-directed RNA polymerase subunit beta 4068948:4072976 forward	2	1 out of 4	1 out of 4
*malT*	DNA-binding transcriptional activator/maltotriose-ATP-binding protein	3	3 out of 4	None
*inaA_ig*	Hypothetical protein BWG_2010	1	1 out of 4	1 out of 4
*rpoA*	RNA polymerase, alpha subunit	1	2 out of 4	1 out of 4
*slyD*	FKBP-type peptidyl prolyl *cis*-*trans *isomerase (rotamase)	4	None	3 out of 4
*xapA*	Purine nucleoside phosphorylase II	2	1 out of 4	1 out of 4

*Present only in *mutY *mutator isolates.

The most striking specific parallel adaptation was a nsSNP in the *rpoA *gene encoding RNA polymerase subunit A. The same base change at the same position was present in three separate populations. The mutation affects a residue in the α-CTD domain of RpoA that is involved in a multitude of regulatory interactions with DNA and transcription factors [47]. This mutation reduces RpoS levels slightly (Figure S2 in Additional file 1) so may have similar benefits to other mutations, such as in *hfq*, that reduce RpoS levels [48]. However, the effect of the *rpoA *mutation, as well as that in *rpoB*, may be on transcription more generally and needs deeper investigation.

There were also some genes that were selected in only one of the two selection conditions. For example, *malT *and *hfq *were selected only in 0.1 h^-1 ^cultures, while the *slyD *gene encoding an FKBP-type peptidyl prolyl *cis*-*trans *isomerase was selected in three out of four 0.6 h^-1 ^cultures but absent in slow-growth populations. The significance of *slyD *under this selection condition has not been investigated and future studies will need to investigate these and other less understood mutations, as in *inaA *(which may affect a hypothetical protein BWG-2010) and *rpoA*.

Mutations in *malT *cause elevated levels (7- to 15-fold relative to ancestor) of expression of maltose transporter genes *malG *and *lamB *[49] and, consistent with a previous study [35], mutations in *malT *were observed in three out of four populations. We did not find any isolates with high levels of *malG-lacZ *activity in the 160 isolates from 0.6 h^-1 ^cultures. This finding confirms an earlier observation that a *malT*-constitutive mutation is deleterious at high dilution rates [35]. Previously, we showed that a mutation in *hfq *was responsible for the multiple benefits under glucose limitation through changing at least five regulation targets [48]. Mutations in *hfq *exhibit a characteristic phenotype of increased sensitivity to methyl a-glucoside (α-MG) and a slightly elevated level of *malG-lacZ *activity [48]. α-MG sensitive isolates were found in six out of the eight surveyed populations, although the abundance of such isolates varied widely between populations (Table S1 in Additional file 1). To test if *hfq *mutations were responsible for α-MG sensitivity, we sequenced *hfq*, including its transcription region, in all α-MG sensitive isolates. We found an additional 19 *hfq *isolates, but none of them were from 0.6 h^-1 ^cultures. The α-MG-sensitive mutants in fast dilution rate populations were not altered in *hfq *and were probably *mlc *or *ptsG *mutations [35]. The mutations in *hfq *were beneficial at slow growth rates but deleterious at the fast growth rate, explaining why they were not found in 0.6 h^-1 ^cultures [50]. This bias in selection of pleiotropic *malT *and *hfq *could be a contributing factor to the observed difference in phenotypic diversity between 0.1 h^-1 ^and 0.6 h^-1 ^populations.

### A mechanism of mutational divergence and adaptive convergence

Having determined the diverse phenotype, genotype and fitness properties of isolates, we next investigated whether the underlying mechanism(s) leading to the observed diversity could be identified. A recent mutation-selection balance model [16,51] suggested that adaptive mutations with small fitness effects are able to fix only in combination with those of larger effects; independent mutations with small effects were thought to be suppressed as a result of clonal interference, particularly in large populations [52]. Applying this multiple mutation model, one would expect that later-arising isolates should contain beneficial mutations on top of previously accumulated beneficial mutations. By contrast, the convergent evolution mechanism proposed that multiple mutational pathways impacting on the same physiology could generate and maintain diversity within the evolving populations [7,17].

**Figure 5:**
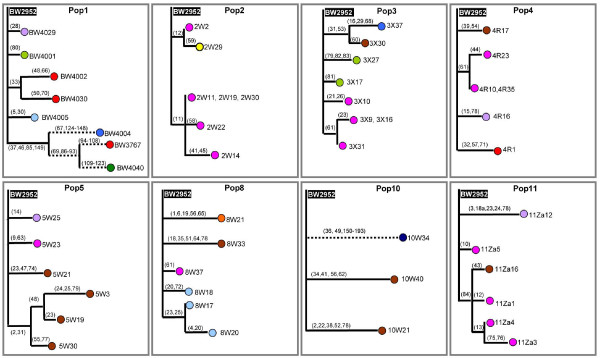
**The multiple pathways of mutational adaptation within evolving *E. coli *populations**. The dendrograms were constructed based on the mutations identified by whole genome resequencing using the maximum parsimony method in PAST [69]. The trees are rooted using the ancestral genome, BW2952 (black box with white type), as out-group. The mutational events (numbers in parentheses) in dendrograms that distinguish the evolved isolates are labeled with numbers in Table S4 in Additional file 1. Color coding of nodes in the trees is indicative of PC groupings. For detailed information about the mutational events see Table S4 in Additional file 1.

We tested these notions in two ways. Firstly, if mutation-selection balance is important, all the evolved isolates within the same population should have originated from the most-fit initial clone. As found earlier, frequently the most-fit isolates harbored mutations in *rpoS*, which according to the previous studies using the same population, initially were present in all eight populations [35]. Therefore, we expected all evolved isolates to contain an *rpoS *mutation. However, contrary to the model, our maximum likelihood phylogenetic analysis showed that multiple lineages emerged independently in all populations and coexisted with the *rpoS *subpopulations (Figure 5). For example, mutations in *rpoA, hfq *and *rssB *occurred independently in the ancestor in population 3, separately from the *rpoS *isolates. In all eight populations, multiple beneficial mutations routinely accumulated in competing lineages, rarely resulting in the complete clonal replacement of subpopulations. These results suggest clonal interference is not strong enough to prevent eventual accumulation of competitors, as was also found in evolving asexual yeast populations [53].

**Figure 6:**
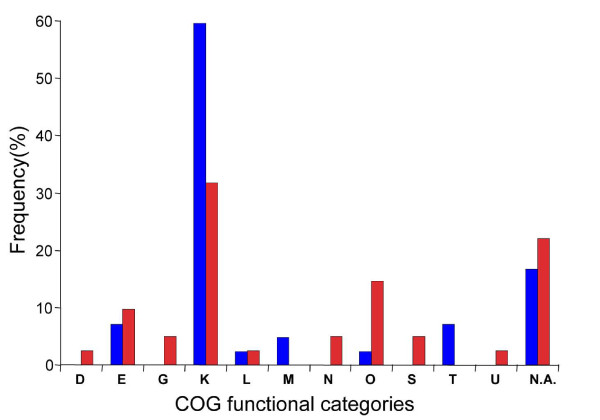
**Functional changes in 85 mutations identified in 44 non-mutator isolates from 4 slow-growing cultures and 4 fast growing cultures**. Groupings of genes were based on COGs (Clusters of Orthologous Groups) [74]. COG codes: D, cell cycle control and mitosis; E, amino acid metabolism and transport; G, carbohydrate metabolism and transport; K, transcription; L, replication and repair; M, cell wall/membrane/envelope biogenesis; N, cell motility; O, post-translational modification, protein turnover, chaperone functions; S, function unknown; T, signal transduction; V, intracellular trafficking and secretion; N.A., not analyzed.

Secondly, we investigated the function of mutated genes within each population to test whether our results fit with the convergent evolution proposal [7,17]. As shown in Figure 6, most of the 85 mutations in Table S4 in Additional file 1 (about 55% in 0.1 and 30% in 0.6 h^-1 ^populations) were located in genes with regulatory functions according to COG (Cluster of Orthologous Groups) functional categories. These results confirm the earlier studies by Conrad *et al*. [21] and Barrick *et al*. [41], that regulatory mutations provide a main route of mutational adaptation. Mutations in *rpoA, rpoB, csgD, rssB *and *fhlA *as well as previously identified mutations in regulatory genes *hfq, rpoS, mglD *(*galS*) and *malT *were the most frequent (Table 2; Table S4 in Additional file 1). In addition to controlling glucose transport, several of these genes are involved in resetting the trade-off between stress protection and nutritional competence (abbreviated as SPaNC [34]). Mutations in *csgD, rssB, cyaA, hfq *and *rpoS*, as well as possibly *rpoA and rpoB*, affect regulation of the general stress response directly or indirectly. Mutations in *rpoS *were the most common, appearing in six out of eight populations. This gene directly, and the others indirectly, influence the SPaNC balance [13,34]. Hence, an altered SPaNC trade-off, which can allow co-existence of multiple types [13,34], is a common event in the eight populations.

Newly found mutations affecting the SPaNC balance include two distinct mutations, a SNP and an IS*1 *insertion, in the transcribed message of *csgD *in isolates from three populations. The *csgD *gene encodes a DNA-binding transcription activator that regulates several genes involved in biofilm formation and also influences σ^S ^[54,55]. Also, in addition to a previously identified mutation in *hfq *[48], we found two further alleles of *hfq *at slow growth rates affecting translation of σ^S ^. We also found one change in *rssB *relative to the ancestor; RssB facilitates and regulates degradation of σ^S ^by the protease ClpXP under nutrient-sufficient conditions [56]. RssB can also inhibit σ^S ^function even in the absence of proteolysis, possibly via stoichiometric binding [56]. An immuno-blot analysis with an anti-σ^S ^serum also indicated that these *csgD, rssB *and *hfq *isolates indeed contain lowered σ^S ^levels (Figure S2 in Additional file 1), suggesting that retuning of the SPaNC trade-off may be the common consequence of the different regulatory mutations. The convergent evolution through diverged mutational pathways has also been identified in *E. coli *populations during thermal adaptation [57].

## Discussion

A combination of phenotypic and genotypic analysis has revealed the considerable extent of diversity that develops in eight glucose-limited chemostat populations. A simple phenotypic screen revealed 11 PC groups with 10 sharing similar properties across more than one population. The genomic diversity was even greater, because several mutational means of achieving the same phenotype were possible within PC groups. Only 2 out of 48 isolates had the same combination of mutations, revealing the extent of diversity present in glucose-limited *E. coli *populations. Even strains from the same PC group and the same population generally contained many mutational differences; our limited sampling means we have not covered all possibilities. We have identified many new mutations in addition to those that have been identified in earlier studies of glucose-limited populations (*rpoS, ptsG, mglD, malT, hfq*) [48,58]. Especially intriguing are potential global regulatory mutations in *rpoA, csgD*, and *rssB *as well as mutations in *lpx *genes, *fimA, frvB *and *ptsP *affecting membranes and surface functions. These mutations will be individually studied for fitness and physiological effects in future studies. The main emphasis in this discussion though is to discuss the impact of these results on the previously identified diversification mechanisms and then the novel differences in evolutionary paths between bacteria growing at two growth rates, especially to explain the greater phenotypic diversity at the slower growth rates.

### How the new data fit with mechanisms explaining diversity and evolutionary coexistence

Some mechanisms of diversification involve ecological interactions exhibited as frequency-dependence [11]. This includes niche creation [11,12] and frequency-dependent coexistence due to cross-feeding, where the cross-feeder organism is maintained in balance with the producer [59], or through cannibalism of one or the other coexisting type [28]. Another frequency-dependent scenario is when two or more resources in a system result in specialization of different bacteria on different resources in the same environment [11,60]. None of these ecological scenarios are readily extended to the current studies. Cross-feeding and cannibalism do not appear to have evolved in the chemostats, probably because the time-scale is relatively short. None of the sequenced mutations hint at cross-feeding, such as the *acs *mutation noted in [8]. There is no evidence for multiple resources, multiple niches or mutations enhancing uptake or regulation of alternative carbon sources besides glucose. Nevertheless, isolates that are less fit than the ancestor at a 50:50 population ratio do show negative frequency dependence, such as BW4005. This PC class is a minority in the 0.1 h^-1 ^populations and absent from the 0.6 h^-1 ^populations, so frequency-dependent selection is present. At the moment we have no ecological or mechanistic explanation of the frequency dependence or the mutations in BW4005. Likewise, the mutations in 2W2, 3X16, 3X31, 4R10, and 4R18 do not indicate the frequency-dependence mechanism responsible. Perhaps ecological interactions are not essential for frequency dependence, which may be a manifestation of internal metabolic or regulatory differences. Recent studies suggest the appearance of frequency-dependence can be due to complex fitness-growth rate relationships determined by altered patterns of gene expression [50]. Further investigation is needed to determine whether the mutations in the frequency-dependent isolates show non-linear fitness-growth rate relationships.

The diversity in an evolving population is dependent on the rate of appearance of mutations and the elimination of mutations through selection of the fittest types. Mutation-selection balance is therefore a potential contributor to the coexistence of many types [15,16,51]. The genomics indicates that in a large population such as ours with 10^10 ^bacteria, multiple mutational lineages arise directly from the ancestor; *hfq, rpoA, rpoS *mutations and other co-existing mutations in various populations do not appear on a sequential basis and clonal inference is not sufficient to eliminate the contending lineages. This is in agreement with the model for events in a large population [53,61]. If mutation-selection balance drives diversity, the mutation rate difference between slow- and fast-growing populations should contribute to greater diversity at 0.1 h^-1^. A difference in diversity was indeed seen, but the problem is that the fitness properties of the isolates are also different. An even more dramatic case of altered mutation supply is with the *mutY *mutator sub-populations in two populations, with ten times as many mutations as the rest of the isolates. Contrary to the expectation that these high mutation rate lineages should diversify more readily by mutation selection balance, we do not see this result. Indeed, in time points subsequent to the one studied here, the mutator sub-populations do not diversify or take over populations (Figure S1 in Additional file 1). So overall, firm conclusions on mutation-selection balancing as a factor in chemostat populations are not possible, but it is unlikely to be the dominant contributor to the observed divergence.

Trade-offs in metabolism [33,62], rate-yield [63,64], and stress-nutrition [13,34] have all been postulated to lead to alternative evolutionary solutions, even in uniform environments. More recently, combinations of trade-offs have been modeled [10] to explain the level of diversity seen in chemostat populations. Several trade-offs indeed change in chemostat evolution and result in isolates with altered stress resistance-nutrition properties as well as changes in growth rate and yield [33,65]. Even more recently, trade-offs with differences in mutational robustness rate have also been postulated to lead to diversity in evolving populations [14]. Since all of the proposed ingredients (trade-offs, mutation rate differences) are present in chemostat populations as demonstrated in this study, our results are not inconsistent with these proposals. A mathematical model of the SPaNC trade-off, which is being altered by mutation in these populations, does suggest that coexistence of types with distinct settings of the SPaNC balance can be demonstrated [13]. The various *hfq, rssB, rpoS *and possibly *rpoA *mutants do offer alternative settings of the SPANC balance, so may contribute to heterogeneity by providing alternative SPaNC settings. The characterization of the settings and experimental coexistence studies require further work, but trade-offs do offer possibilities for heterogeneity in the eight populations. This is not a blanket explanation, however, because there are isolates in all populations that do not have changes to RpoS levels and SPaNC balances. Here again, this is a partial explanation of diversity.

Finally, a simple explanation of coexistence is the convergent evolution of genetically distinct mutants that have achieved a comparable level of fitness. A particular level of fitness (as measured against ancestor) could be reached in strains having entirely different genomic changes (Figure 4). Bacteria exhibiting alternative regulatory and metabolic pathways and mutational degeneracy in these pathways can lead to co-existing organisms in nutrient-limited chemostat populations [7,17,33,58]. As already noted previously, mutations in *hfq *and *rpoS *can both reset the stress-nutrition trade-off towards better nutrient uptake [17]. Furthermore, in line with a recent study [57], the presence of mutations in *rpoA, csgD, rssB *and *fimA*, each in different members of the same PC group, indicates that there are degenerate means of evolving similar properties within a complex regulatory network. These changes can also overlap with the trade-offs discussed above, so alternative SPaNC settings can be arrived at in degenerate ways.

### The effects of a difference in growth rate in the selection environment

It is instructive to compare adaptational events at two chemostat dilution rates but with otherwise identical ancestors, conditions and sole resources in the shape of glucose. As evident in PCs and with mutations, an adaptive advantage at one growth rate does not necessarily provide an advantage under both growth conditions. There was already evidence that *malT*-con mutations are deleterious at faster growth rates [35], and this study confirms this, as no such mutations were selected in any of the fast-growth populations. It is not clear why *malT *mutations are deleterious at faster growth rates, but the finding of *malT*-negative mutations in batch culture selection with glucose [30] suggests that there is a negative effect of MalT on fitness when growing fast on glucose.

Another example of a gene with mutations beneficial and selected at 0.1 h^-1 ^but not at 0.6 h^-1 ^is *hfq*. All three evolved alleles of this gene provided a benefit at 0.1 h^-1^, which occurs through multiple pleiotropic effects [48]. The mutations were found to be deleterious at 0.6 h^-1 ^[50], explaining their absence from 0.6 h^-1 ^cultures. This fitness difference is likely to be because global gene expression alters with different dilution rates [66] and the role of Hfq changes. We postulate that *hfq *mutations, through numerous epistatic effects, are beneficial when cells are more highly stressed (at 0.1 h^-1^) than when growing faster (at 0.6 h^-1^).

Fitness in evolved isolates is by no means uniform in coexisting types or at different growth rates. The fitness distributions as well as the diversity differ between slow- and fast-growth populations. The high proportion of regulatory mutations observed in the 0.1 h^-1 ^but not the 0.6 h^-1 ^population (Figure 6) is consistent with a different global-regulatory background and demonstrates differences in gene expression between the two conditions [66]. The greater phenotypic diversity in the 0.1 h^-1 ^cultures may well reflect a greater range of regulatory mutations observed at the slower growth rate.

## Conclusions

*E. coli *has at its disposal a redundant set of fitness strategies under glucose limitation involving various combinations of mutations. These permit concurrent evolution of distinct lineages within the same population. The simplest explanation is that fitness arose through multiple lineages of beneficial mutations that did not fully eliminate competitors and invariably led to diversification within the eight clonal cultures. The kind of mutants and level of diversity were influenced by the growth rate of cultures. In total, our results suggest that large bacterial populations are a diverse collection of clonal lineages in most settings, complex or otherwise. Therefore, it is likely that in a natural habitat with a large population size, bacteria develop enormous sympatric variation. This is likely to be of considerable importance in clinical populations of bacteria that exhibit diversification within the lifetime of an infection [3].

## Materials and methods

### Bacterial strains and growth conditions

For the experimental evolution, the *E. coli *K-12 strain BW2952, an MC4100 derivative, was propagated at growth rates (dilution rates) of 0.1 h^-1 ^and 0.6 h^-1 ^in a glucose-limited chemostat exactly as described in [35]. Individuals from the culture were isolated and propagated as in [6]. At least 39 randomly picked isolates (colonies) from days specified in the text were obtained by streaking chemostat samples on nutrient agar.

### Phenotypic assays, classification of phenotypic properties, cluster analysis

The changes in chemostat isolates resulting in *rpoS *mutations were detected by iodine staining as previously described [67] and confirmed by growth tests on acetate plates [65]. The method for assaying *malG-lacZ *fusion b-galactosidase activity for *mal *regulation was described in [67]. The growth conditions for the sensitivity assay for methyl a-glucoside (α-MG) were as described in [68]. Sensitive isolates had an inhibition zone of over 2.5 cm compared to the resistant ancestral strain BW2952.

We then used these triple-phenotypic test results to classify evolved isolates using the criteria shown in Table S2 in Additional file 1. We related differences in the phenotypic properties of *E. coli *chemostat isolates by using the neighbor-joining method of tree construction based on distance estimated by using PAST [69].

### Genome re-sequencing

Chromosomal DNA was prepared as previously described [36]. Libraries for Illumina paired-end sequencing were prepared using the protocols provided by Illumina. A Solexa Genome Analyzer IIx (Illumina, Little Chesterford, Essex, UK) was used to sequence each isolate to a depth of between 96- and 159-fold coverage. The 101 bp Solexa reads generated, after removal of the duplication, were mapped to the *E. coli *BW2952 genome [GenBank:NC_012759] to generate the assembly using BWA [70] with the default parameter, which allows 4% mismatches. The Smith-Waterman algorithm was used to rescue the unmapped end. All the reads with extremely large or small insert size (< 20% or > 200% of normal) were mapped again using BLASTn with an e-value of 0.00001 and the -F F flag. Only the read pairs mapped to a proper insert length and with at least one end of the pairs mapped to non-repeat regions were taken into account. For positions in doubt, due to sequence coverage reduction and in repeat regions, Sanger sequencing was used to resolve the sequence. SAMtools [71] was used to calculate the per-position coverage and base calls for each position.

SNPs in the sequenced strain were called where a position was covered by at least ten reads with at least 80% of the covering reads showing the same mismatch. Other suspected positions were sequenced using Sanger methodology.

Small indels were identified from the alignment between reads and the reference genome. Cases where an indel occurred within homopolymer tracts were manually examined, as BWA and BLASTn alignment can place such indels at different positions along the homopolymer. Indels in homopolymers are not subject to elevated error or bias in Solexa sequencing [72]. Therefore, small indels could be reliably resolved by the set of individual Solexa reads spanning the homopolymer region.

Large insertion, duplication or deletion events were also detected from the alignment between reads and the reference genome. Deletions were determined as regions that were not covered by any single read and flanked by read pairs with extremely large insert size. The positions of the large insertions or duplications, such as IS movement or copy number variation in the sequenced genome relative to the reference genome, could be detected as read pairs aligned around the insert positions that have one of their ends unaligned or aligned to other regions that represent the beginning or end of the inserted sequence.

### Estimation of diversity index

Simpson's diversity index (*D*) was determined using the following equation:

$$Di=\sum {\left(\text{n}/\text{N}\right)}^{2}$$

where 'n' is the total number of isolates of a particular phenotype and 'N' is the total number of isolates of all phenotypes.

### Pair-wise competition experiments and estimation of selection coefficients (S) and estimation of fitness

To estimate the selection coefficient, each evolved isolate was competed with the *metC*::Tn10 derivative of the ancestral strain (BW2952) in the same conditions in which isolates had evolved. In each case, both evolved isolates and reference strains were grown independently overnight in glucose-limited chemostats in modified Mcbride agar (MMA) medium supplemented with 0.02% (w/v) glucose plus 4 μg/ml methionine to ensure they were in the same physiological conditions as well as reacclimatize the selection condition. The selection coefficient (S) of evolved isolates was estimated using at least three time points with R^2 ^≥ 0.95 as previously described in [6]. The selection coefficient for evolved isolates was determined from the slope of the linear regression of ln[p(t)/q(t)] against at least three time points [73], where p(t) and q(t) are the proportions of two competing strains at time t hours. Since, competition experiments were performed under two different dilution rates, the fitness of evolved isolates is expressed as the selection per generation, Sg (calculated as (ln 2)S/D, where D is dilution rate) [73].

### Cluster analysis

The dendrograms were constructed based on mutations identified by whole genome resequencing using the maximum parsimony method in PAST [69].

### Estimation of mutation accumulation rates

MARs were estimated using the following equation as described in [52]:

$$\text{MAR}=k/\left(N_{\text{e}}.2Sg.T\right)$$

where *k *is the average number observed mutations, *T *is the number of generations, *N*_e _is the effective population size and *Sg *is the selection coefficient per generation.

### Data availability

The sequencing data generated in this study have been deposited at the DNA Data Bank of Japan under accession number DRA000563.

## Abbreviations

α-MG: methyl a-glucoside; bp: base pair; COG: Cluster of Orthologous Groups; IS: insertion sequence; MAR: mutation accumulation rate; nsSNP: non-synonymous SNP; PC: phenotypic class; SNP: single nucleotide polymorphism; SPaNC: stress protection and nutritional competence.

## Competing interests

The authors declare that they have no competing interests.

## Authors' contributions

RM carried out the phenotypic studies, as well as analyses of fitness in chemostats and mutation effects. TF conceived of the study, and participated in its design and coordination and wrote the draft manuscript. PR was involved in data analysis and paper writing. YL and BL carried out sequencing and analysis. LW participated in the design of the study and coordinated the genome sequencing and data analysis. All authors read and approved the final manuscript.

## Supplementary Material

Additional file 1 **Supplementary Tables S1 to S4 and Figures S1 and S2**. Table 1: phenotypic characteristics of 320 *E. coli *isolates evolving under glucose-limited limited. Table 2: groupings of phenotypic properties found in eight glucose-limited populations of *E. coli*. Table 3: clones chosen for full genome resequencing. Table 4: genomic changes detected in the 44 non-mutator evolved clones. Figure 1: maintenance of phenotypic diversity in an *E. coli *population evolving in a glucose-limited chemostat. Figure 2: relative level of RpoS protein in glucose-limited chemostats. Click here for file

Additional file 2 **SNPs of 48 strains compared with BW2952**. Sheet 1: SNPs of slow-growth isolates. Sheet 2: SNPs of fast-growth isolates. Sheet 3: the SNPs of isolates with *mutY *mutator. Click here for file
