# Supplementary material for: The multiplicity of divergence mechanisms in a single evolving population
Source: Genome Biol. 2012 Jun 8;13(6):R41. doi: 10.1186/gb-2012-13-6-r41 (PMC3446313; doi:10.1186/gb-2012-13-6-r41)
Supplement: Additional file 1 — Supplementary Tables S1 to S4 and Figures S1 and S2. Table 1: phenotypic characteristics of 320 E. coli isolates evolving under glucose-limited limited. Table 2: groupings of phenotypic properties found in eight glucose-limited populations of E. coli. Table 3: clones chosen for full genome resequencing. Table 4: genomic changes detected in the 44 non-mutator evolved clones. Figure 1: maintenance of phenotypic diversity in an E. coli population evolving in a glucose-limited chemostat. Figure 2: relative level of RpoS protein in glucose-limited chemostats. [file gb-2012-13-6-r41-S1.DOC]

**The multiplicity of divergence mechanisms in a single evolving population**

Ram P. Maharjan^1^, Thomas Ferenci^1^, Peter R.Reeves^1^, Yang Li^2,3^ ,Bin Liu^2,3^, and Lei Wang*^2,3^

**SUPPLEMENTARY INFORMATION**

**Table S1. Phenotypic characteristics of 320 *E. coli* isolates evolving under glucose-limited limited chemostats**

**Legend to Table S1:**

Pop1-4 were obtained from slow or D=0.1 h^-1^ dilution rate cultures.

Pop5, 8, 10 and 11 were obtained from fast or D=0.6 h^-1^ dilution rate cultures.

Iodine staining: -, no staining; P, partial brown stain, +, dark brown stain

*malG*-*lacZ* expression value: presented as relative to wild-type; wild-type strain fusion activity is around 100 Miller Units.

Phenotypic classifications (PCs) were based on the criteria shown in Table S2.

**Table S2** Groupings of phenotypic properties found in eight glucose-limited populations of *E. coli*

| **Class** | **Iodine^1^** | **α-MG sensitivity^2^** | **β-galactosidase activity^3^ (fold-change relative to ancestor)** |
| --- | --- | --- | --- |
| **Ancestor** | + | R | 1 |
| 1 | + | R | 1 |
| 2 | + | R | 2 |
| 3 | P | R | 2 |
| 4 | P | S | 2 |
| 5 | P | S | 4 to 6 |
| 6 | - | R | 1 |
| 8 | - | R | 2 |
| 7 | - | S | 2 |
| 9 | - | R | 7 to 9 |
| 10 | - | R | 10 to 15 |
| 11 | - | S | 10 to 15 |

^1^The categories of phenotypes found were: +, wild-type staining with iodine and slow growth on acetate: -, staining and fast growth on acetate characteristic of *rpoS* null mutants; P, partial staining and intermediate growth rates on acetate.

^2^The categories of phenotypes found were: R, no zone of inhibition, as found with ancestor BW2952; S, sensitive to α-MG with zones of inhibition > 2.5 cm.

^3^The β-galactosidase (*malG-lacZ*) activity ranges found for classes of isolates is given.

**Table S3.** List of clones chosen for full genome resequencing

| **Strains** | **Population** | **Phenotypic Class** | **Dilution rate (h^-1^)** | **Age of culture (days)** |
| --- | --- | --- | --- | --- |
| BW3767 | 1 (1Za) | PC10 | 0.1 | 26 |
| BW4001 | 1 (1Za) | PC5 | 0.1 | 26 |
| BW4002 | 1 (1Za) | PC10 | 0.1 | 26 |
| BW4004 | 1 (1Za) | PC9 | 0.1 | 26 |
| BW4005 | 1 (1Za) | PC2 | 0.1 | 26 |
| BW4029 | 1 (1Za) | PC1 | 0.1 | 26 |
| BW4030 | 1 (1Za) | PC9 | 0.1 | 26 |
| BW4040 | 1 (1Za) | PC11 | 0.1 | 26 |
| 2W2 | 2 (2W) | PC3 | 0.1 | 23 |
| 2W11 | 2 (2W) | PC3 | 0.1 | 23 |
| 2W14 | 2 (2W) | PC3 | 0.1 | 23 |
| 2W30 | 2(2W) | PC3 | 0.1 | 23 |
| 2W22 | 2 (2W) | PC3 | 0.1 | 23 |
| 2W29 | 2 (2W) | PC4 | 0.1 | 23 |
| 3X9 | 3 (3X) | PC3 | 0.1 | 24 |
| 3X10 | 3 (3X) | PC3 | 0.1 | 24 |
| 3X16 | 3 (3X) | PC3 | 0.1 | 24 |
| 3X17 | 3 (3X) | PC5 | 0.1 | 24 |
| 3X27 | 3 (3X) | PC5 | 0.1 | 24 |
| 3X30 | 3 (3X) | PC8 | 0.1 | 24 |
| 3X31 | 3 (3X) | PC3 | 0.1 | 24 |
| 3X37 | 3 (3X) | PC9 | 0.1 | 24 |
| 4R1 | 4 (4R) | PC10 | 0.1 | 18 |
| 4R10 | 4 (4R) | PC3 | 0.1 | 18 |
| 4R16 | 4 (4R) | PC1 | 0.1 | 18 |
| 4R17 | 4 (4R) | PC3 | 0.1 | 18 |
| 4R23 | 4 (4R) | PC3 | 0.1 | 18 |
| 4R18 | 4 (4R) | PC3 | 0.1 | 18 |
| 5W3 | 5 (5W) | PC8 | 0.6 | 23 |
| 5W19 | 5 (5W) | PC8 | 0.6 | 23 |
| 5W21 | 5 (5W) | PC8 | 0.6 | 23 |
| 5W23 | 5 (5W) | PC3 | 0.6 | 23 |
| 5W25 | 5 (5W) | PC1 | 0.6 | 23 |
| 5W30 | 5 (5W) | PC8 | 0.6 | 23 |
| 8W17 | 8 (8W) | PC2 | 0.6 | 23 |
| 8W18 | 8 (8W) | PC2 | 0.6 | 23 |
| 8W20 | 8 (8W) | PC2 | 0.6 | 23 |
| 8W21 | 8 (8W) | PC7 | 0.6 | 23 |
| 8W33 | 8 (8W) | PC8 | 0.6 | 23 |
| 8W37 | 8 (8W) | PC3 | 0.6 | 23 |
| 10W21 | 10 (10W) | PC8 | 0.6 | 23 |
| 10W34 | 10 (10W) | PC6 | 0.6 | 23 |
| 10W40 | 10 (10W) | PC8 | 0.6 | 23 |
| 11Za1 | 11 (11Za) | PC3 | 0.6 | 26 |
| 11Za3 | 11 (11Za) | PC3 | 0.6 | 26 |
| 11Za4 | 11 (11Za) | PC1 | 0.6 | 26 |
| 11Za5 | 11 (11Za) | PC3 | 0.6 | 26 |
| 11Za12 | 11 (11Za) | PC1 | 0.6 | 26 |
| 11Za16 | 11 (11Za) | PC8 | 0.6 | 26 |

**Table S4.** Genomic changes detected in the 44 non-mutator evolved clones

| **Event no.** | **Position^a^** | **Gene or location^b^** | **Product** | **COG^c^** | **Ancestral nucleotide** | **Change to** | **Type** | **Present in (clones)** | **Selection conditions dilution rate (h^-1^)** |
| --- | --- | --- | --- | --- | --- | --- | --- | --- | --- |
| 1 | 98536 | *ftsW* | Integral membrane protein involved in stabilizing FstZ ring during cell division | D | T | G | nsSNP | 8W21 | 0.6 |
| 2 | 142735 | *yadG_ig* | ATP-binding component of ABC superfamily | V | A | C | iSNP | 5W3, 5W19, 5W30, 10W21 | 0.6 |
| 3 | 456321 | ppiB | peptidyl-prolyl cis-trans isomerase B (rotamase B) | O | C | T | nsSNP | 11Za12 | 0.6 |
| 4 | 526598 | fepB-ig | ron-enterobactin transporter subunit | N.A. | A | * | Del | 8W20 | 0.6 |
| 5 | 541030 | ahpC | subunit of alkylhydroperoxide reductase | O |  | * | Del | BW4005 | 0.1 |
| 6 | 862349 | *ycaL_ig* | Putative peptidase with chaperone function | O | T | * | Del | 8W21 | 0.6 |
| 7 | 871695 | ycaQ | conserved protein | S | C | T | nsSNP | 10W40 | 0.6 |
| 8 | 999983 | *ycdU_serX_ig* | NA | N.A. | * | 546bp | Ins | 11Za3 | 0.6 |
| 9 | 1006354 | *csgD_ig* | DNA-binding transcriptional activator in two-component regulator | KT | C | T | nsSNP | 5W23 | 0.6 |
| 10 | 1006521 | *csgD_ig* | DNA-binding transcriptional regulator | KT | A | G | nsSNP | 11Za5 | 0.6 |
| 11 | 1006550 | *csgD_ig* | DNA-binding transcriptional activator in two-component regulator | KT | * | IS1 | Ins | 2W22, 2W14, 2W11, 2W19, 2W30 | 0.1 |
| 12 | 1006579 | *csgD_ig* | " | KT | * | IS1 | Ins | 2W2, 2W29,11Za1 | 0.1 |
| 13 | 1007442 | *csgB* | Curlin, minor subunit precursor | NU | * | IS1 | Ins | 11Za3, 11Za4 | 0.6 |
| 14 | 1007881 | *csgA* | cryptic curlin major subunit | S |  | IS1 | Ins | 5W25 | 0.6 |
| 15 | 1018151 | mdtG-lpxL _ig |  | N.A. | * | IS5 | Ins | 4R16 | 0.1 |
| 16 | 1019466 | *lpxL* | Lauryl-acyl carrier protein (ACP)-dependent acyltransferase | M | C | A | nsSNP | 3X37 | 0.1 |
| 17 | 1061050 | *ptsG* | PTS system glucose-specific transporter subunits IIBC | G | G | C | nsSNP | 8W21 | 0.6 |
| 18 | 1073853 | mfd | transcription-repair coupling factor | LK | A | C | nsSNP | 8W33 | 0.6 |
| 18a | 1107442 | unknownIg | uncharactrised region | N.A. |  | IS1 |  | 11Za12 | 0.6 |
| 19 | 1107638 | unknown | unknown | N.A. | * | IS1 | Ins | 8W20 | 0.6 |
| 20 | 1108551 | unknown | unknown | N.A. | * | IS1 | Ins | 8W18 | 0.6 |
| 21 | 1178207..1178975 | *rssB* leader | response regulator of RpoS | T | 769bp | * | Del | 3X10 | 0.1 |
| 22 | 1181955 | INS (*hns*) | *hns* transcription unit | N.A. | * | IS1 | Ins | 10W21 | 0.6 |
| 23 | 1191049 | *BWG_1070_ig* | Predicted divalent heavy-metal cations transporter | N.A. | C | * | Del | 3X9, 3X16, 5W19, 5W21, 8W17, 8W20,11Za12 | 0.1/0.6 |
| 24 | 1288806 | DEL (*fnr*-insH) | between *fnr* and insH may affect frn expression | N.A. | C | * | Del | 5W3, 10W40, 11Za12 | 0.6 |
| 25 | 1289365 | DEL | between *fnr* and insH may affect frn expression | N.A. | G | * | Del | 5W3, 8W17, 8W20 | 0.6 |
| 26 | 1815385 | holE | DNA polymerase III, theta subunit | T | T | A | nsSNP | 3X10 | 0.1 |
| 27 | 1829555 | *lpxM* | Lipid A biosynthesis (KDO)2-(lauroyl)-lipid IVA acyltransferase | M | C | A | nsSNP | BW4005 | 0.1 |
| 28 | 1887607 | *yecC* | ATP-binding component of ABC superfamily | E | G | T | nsSNP | BW4029 | 0.1 |
| 29 | 2056289 | *yegQ* | Predicted peptidase | O | C | A | nsSNP | 3X37 | 0.1 |
| 30 | 2058010 | *ogrK-yegS* |  | N.A. |  | * | Del | BW4005 | 0.1 |
| 31 | 2131125 | *mglO* | DNA binding transcriptional regulator | K | G | A | iSNP | 3X37, 3X30, 5W3, 5W19, 5W30 | 0.1/0.6 |
| 32 | 2131126 | *mglO* | DNA binding transcriptional regulator | K | A | * | Del | 4R1 | 0.1 |
| 33 | 2131130 | *mglO* | " | K | C | A | iSNP | BW4002, BW4030 | 0.1 |
| 34 | 2131134 | *mglD* | DNA-binding transcriptional repressor | K | T | G | nsSNP | 10W40 | 0.6 |
| 35 | 2131136 | *mglD* | DNA-binding transcriptional repressor | K | C | T | nsSNP | 8W33 | 0.6 |
| 36 | 2131326 | *mglD* | DNA-binding transcription regulator | K | G | T | nsSNP | 10W34 | 0.6 |
| 37 | 2131806 | *mglD* | “ | K | G | T | nsSNP | BW3767, BW4004, BW4040 | 0.1 |
| 38 | 2132010 | *mglD* |  | K | * | IS2 | Ins | 10W21 | 0.6 |
| 39 | 2132180-90 | *mglO* | DNA binding transcriptional regulator | K | 11bp | * | Del | 4R17 | 0.1 |
| 40 | 2168261 | *yejM* | Putative hydrolase, inner membrane | R | A | T | nsSNP | BW4002 | 0.1 |
| 41 | 2233448 | inaA_ig | hypothetical protein BWG_2010 | N.A. | A | C | iSNP | 2W14, 10W40 | 0.1/0.6 |
| 42 | 2408668 | xapA | Purine nucleoside phosphorylase II | E | C | A | nsSNP | BW4030 | 0.1 |
| 43 | 2408239 | xapA | Purine nucleoside phosphorylase II | E | C | T | nsSNP | 11Za16 | 0.6 |
| 44 | 2691767 | proX | Glycine betaine transporter subunit | E | A | C | nsSNP | 4R23 | 0.1 |
| 45 | 2739814 | fhlA | DNA-binding transcriptional activator | KT | A | C | nsSNP | 2W14 | 0.1 |
| 46 | 2750443 | *rpoS* | RNA polymerase sigma fsubunit | K | C | A | nsSNP | BW3767, BW4004, BW4040 | 0.1 |
| 47 | 2750539 | *rpoS* | " | K | A | C | nsSNP | 5W21 | 0.6 |
| 48 | 2750685 | *rpoS* | " | K | A | T | nsSNP | BW4002 | 0.1 |
| 49 | 2750812 | *rpoS* | " | K | C | A | nsSNP | 10W34 | 0.6 |
| 50 | 2750814 | *rpoS* | " | K | T | G | nsSNP | BW4030 | 0.1 |
| 51 | 2750982 | *rpoS* | " | K | T | G | nsSNP | 8W33 | 0.6 |
| 52 | 2751003 | *rpoS* | " | K | A | T | nsSNP | 5W3, 5W19, 10W21 | 0.6 |
| 53 | 2751022 | *rpoS* | " | K | C | A | nsSNP | 3X30, 3X37 | 0.1 |
| 54 | 2751102 | *rpoS* | " | K | A | T | nsSNP | 4R17 | 0.1 |
| 55 | 2751160 | *rpoS* | " | K | C | T | nsSNP | 5W30 | 0.6 |
| 56 | 2751325 | *rpoS* | " | K | C | A | nsSNP | 8W21, 10W40 | 0.6 |
| 57 | 2751428 | *rpoS* | " | K | * | IS1 | Ins | 4R1 | 0.1 |
| 58 | 2763405 | ygbT | Conserved protein | L | C | A | nsSNP | 2W22 | 0.1 |
| 59 | 2852015 | *ptsP* | PEP-protein phosphotransferase (enzyme I)/GAF domain containing protein | T | * | T | Ins | 2W29 | 0.1 |
| 60 | 3152313 | *tdcR_ig* | DNA-binding transcriptional regulator | N.A. | C | A | iSNP | 3X30 | 0.1 |
| 61 | 3325374 | *rpoA* | RNA polymerase, alpha subunit | K | C | A | nsSNP | 3X9, 3X16, 3X31, 4R10, 4R23, 4R35, 8W37 | 0.1/0.6 |
| 62 | 3363087 | *slyD* | FKBP-type peptidyl prolyl cis-trans isomerase (rotamase) | O | T | A | nsSNP | 10W40 | 0.6 |
| 63 | 3363584 | *slyD* | FKBP-type peptidyl prolyl cis-trans isomerase (rotamase) | O | C | * | Del | 5W23 | 0.6 |
| 64 | 3362937..3369818 | slyD…. | 9 genes- slyD, kefBG…. | O | 7kb | * | Del | 8W33 | 0.6 |
| 65 | 3363106..3370691 | *slyD_prkB* | 9 genes - slyD, kefBG…. | O | 7.8kb | * | Del | 8W21 | 0.6 |
| 66 | 3439794 | *malT* | DNA-binding transcriptional activator/maltotriose-ATP-binding protein | K | A | G | nsSNP | BW4002 | 0.1 |
| 67 | 3439629 | *malT* | " | K | C | A | nsSNP | BW4004 | 0.1 |
| 68 | 3440085 | *malT* | " | K | T | A | nsSNP | 3X37 | 0.1 |
| 69 | 3440306 | *malT* | “ | K | G | T | nsSNP | BW3767, BW4040 | 0.1 |
| 70 | 3440674 | *malT* | “ | K | C | A | nsSNP | BW4030 | 0.1 |
| 71 | 3440676 | *malT* | “ | K | A | C | nsSNP | 4R1 | 0.1 |
| 72 | 3444633 | rtcR_ig | Intergenic (between rtcB and rtcR) | KT | T | C | nsSNP | 8W18 | 0.6 |
| 73 | 3965948 | yiiD | Predicted acetyltransferase | E | G | * | Del | 10W40 | 0.6 |
| 74 | 3979635 | *frvB* | MG1655 equivalent: b3899 | G | A | C | nsSNP | 5W21 | 0.6 |
| 75 | 4016918 | *metB* | Cystathionine gamma-synthase, PLP-dependent | E | G | T | snsSNP | 11Za3 | 0.6 |
| 76 | 4019113 | *metL* | Fused aspartokinase II/homoserine dehydrogenase II | E | G | * | Del | 11Za3 | 0.6 |
| 77 | 4137859 | BWG_3697 | Protein Kil | N.A. | G | * | Del | 5W30 | 0.6 |
| 78 | 4137860 | *malG*-*lacZ*region | *malG*-fusion region | N.A. | G | * | Del | 4R16, 8W33, 10W21, 11Za12 | 0.1/0.6 |
| 79 | 4337027 | *hfq_ig* | RNA-binding protein | R | * | IS1 | Ins | 3X27 | 0.1 |
| 80 | 4337108 | *hfq* | " | R | T | G | nsSNP | BW4001 | 0.1 |
| 81 | 4337182 | *hfq* | " | R | T | G | nsSNP | 3X17 | 0.1 |
| 82 | 4446676 | *insI (IS3)_ig* | IS186/IS421 transposase | N.A. | G | A | nsSNP | 3X27 | 0.1 |
| 83 | 4446677 | *insI (IS3)_ig* | " | N.A. | G | A | nsSNP | 3X27 | 0.1 |
| 84 | 4480124 | *fimA* | Major type 1 subunit fimbrin | NU | * | IS186 | Ins | 11Za, 11Za3, 11Za4 | 0.6 |

**SUPPLEMENTARY FIGURES**

**Figure S1. Maintenance of phenotypic diversity in *E. coli* population evolving in a glucose-limited chemostat**

The PC groupings of Pop1 isolates were determined in 40 isolates randomly picked at each indicated time point. Phenotypic classifications were based on the characters in Table S2 and the colour coding of PCs is the same as for Fig. 1.

**Figure S2**

Relative level of RpoS protein in glucose-limited chemostats

The evolved isolates with different regulatory mutations were quantitated by immunoassays as in .

Protein samples obtained from stationary phase cultures growing aerobically on L-broth at 37^o^C were run on 12.5% polyacrylamide gels. Proteins RpoD and RpoS were identified as before . Densitometric analysis of western blot images with RpoD and RpoD was performed using ImageJ. Values standardized as described are shown and each is an average of two independent assays, with variation less than 10%.
